# Modulating Spatial Processes and Navigation via Transcranial Electrical Stimulation: A Mini Review

**DOI:** 10.3389/fnhum.2017.00649

**Published:** 2018-01-09

**Authors:** Tad T. Brunyé

**Affiliations:** ^1^Center for Applied Brain and Cognitive Sciences, School of Engineering, Tufts University, Medford, MA, United States; ^2^Cognitive Science Team, U.S. Army Natick Soldier Research, Development and Engineering Center, Natick, MA, United States; ^3^Department of Psychology, Tufts University, Medford, MA, United States

**Keywords:** transcranial direct current stimulation, transcranial alternating current stimulation, spatial cognition, visualization, navigation, functional connectivity

## Abstract

Transcranial electrical stimulation (tES) uses low intensity current to alter neuronal activity in superficial cortical regions, and has gained popularity as a tool for modulating several aspects of perception and cognition. This mini-review article provides an overview of tES and its potential for modulating spatial processes underlying successful navigation, including spatial attention, spatial perception, mental rotation and visualization. Also considered are recent advances in empirical research and computational modeling elucidating several stable cortical-subcortical networks with dynamic involvement in spatial processing and navigation. Leveraging these advances may prove valuable for using tES, particularly transcranial direct and alternating current stimulation (tDCS/tACS), to indirectly target subcortical brain regions by altering neuronal activity in distant yet functionally connected cortical areas. We propose future research directions to leverage these advances in human neuroscience.

## Introduction

Decades of empirical research have demonstrated involvement of diverse lateral and medial brain regions in spatial processing and navigation, including parietal, prefrontal and medial temporal areas (Spiers and Maguire, 2006; Iaria et al., 2007; Whitlock et al., 2008). The reliable involvement of these regions has made them of interest as targets for electrical neuromodulation, attempting to alter the acquisition of spatial knowledge and skills (Brunyé et al., 2014; Wright and Krekelberg, 2014; Oldrati et al., 2018). However, noninvasive electrical neuromodulation is largely limited to superficial cortical layers, limiting the ability to directly target deeper brain structures such as the retrosplenial cortex, posterior cingulate cortex (PCC), or the medial temporal lobes (de Berker et al., 2013). Recent advances in functional connectivity analyses have revealed stable functional networks (Kravitz et al., 2011; Sherrill et al., 2015; Boccia et al., 2017), suggesting that the modulation of superficial brain regions such as the inferior parietal lobule and lateral prefrontal cortex may carry powerful downstream consequences for deeper brain systems involved in spatial processing and real-world navigation. The present mini-review article provides an overview of existing literature using Transcranial electrical stimulation (tES) to modulate several spatial processes underlying navigation behavior, and then proposes that continuing electrical neuromodulation research leverages recent advances in functional connectivity to afford indirect targeting of deep brain areas of critical importance to spatial processing (Keeser et al., 2011; Weber et al., 2014).

## Transcranial Electrical Stimulation

tES is a neuroscientific method for inducing transient alterations in neuronal membrane potential by administering electrical current via electrodes positioned on the scalp (Nitsche et al., 2008; Silva et al., 2008; Woods et al., 2016). Evidence from animal models and computational modeling demonstrates that tES can induce a subthreshold depolarization of pyramidal and possibly glial cells (Ruohonen and Karhu, 2012; Molaee-Ardekani et al., 2013; Rahman et al., 2013), and a growing body of literature demonstrates behavioral impacts of tES on a range of perceptual, cognitive, social and emotional tasks (Jacobson et al., 2012; Santiesteban et al., 2012; Berlim et al., 2013; Brunoni and Vanderhasselt, 2014; Dedoncker et al., 2016). While there are several techniques for administering tES, the present mini-review focuses on the most commonly-used method, transcranial direct current stimulation (tDCS), and incorporates some recent innovations in transcranial alternating current stimulation (tACS; Paulus, 2011; Woods et al., 2016).

With tDCS, low intensity direct current is delivered via electrodes arranged on the scalp. Traditionally, tDCS is delivered in a so-called bipolar montage, involving one anodal and one cathodal electrode, typically positioned directly over a cortical target (Paulus, 2011). For instance, one popular bipolar montage involves placing an anodal electrode over the left dorsolateral prefrontal cortex (dlPFC; 10/20 site F3), with the cathode placed on the right supraorbital area (Brunoni and Vanderhasselt, 2014; Dedoncker et al., 2016). This montage is thought to increase neuronal excitability in the left dlPFC, inducing behavioral effects on working memory and executive control (Tremblay et al., 2014). More recently, multi-electrode montages are being used in an effort to administer relatively focalized stimulation to cortical targets, typically involving 4–5 electrodes arranged in an optimal manner (i.e., higher density at target) according to finite element electrical field models of the human head (Datta et al., 2009; Ruffini et al., 2014).

With tACS, low intensity alternating current is delivered via electrodes arranged on the scalp, in a similar manner to tDCS. However, tACS typically administers sine-wave stimulation waveforms that specifically target frequency bands of cortical oscillations (Herrmann et al., 2013). tACS can thus administer current that is frequency-matched to an intrinsic frequency of a cortical area or network. Computational modeling suggests that tACS may thus be capable of promoting specific activity frequencies in brain areas or networks, perhaps via entrainment (Ali et al., 2013), or plasticity alterations (Vossen et al., 2015). If oscillatory brain activity is fundamental to information processing and behavior, then modulating oscillations with tACS should selectively alter such functions; some recent studies have found promise in this technique (Sejnowski and Paulsen, 2006; Herrmann et al., 2013; Neuling et al., 2013; Chander et al., 2016).

## tES and Spatial Processing

Whereas many tDCS and tACS studies focus on modulating working memory task performance (e.g., Jaušovec et al., 2014; Martin et al., 2014), an emerging body of empirical research has demonstrated some impacts on the spatial processes underlying navigation behavior, including spatial perception and attention, mental rotation, and spatial visualization. This typology of spatial processes generally follows that of Linn and Petersen (1985). Below we report the results of a literature review examining tDCS and tACS influences on spatial processing and navigation, with papers identified via Google Scholar and PubMed, using the terms *tDCS, tACS, spatial cognition, spatial perception, spatial attention, mental rotation, spatial visualization, wayfinding* and/or *navigation*.

### Spatial Attention

Spatial attention involves the dynamic and selective prioritization and sustainment of attention toward locations in space (Posner, 1980). A number of distributed brain areas have been implicated in spatial attention, most notably the posterior parietal cortex (PPC) in a primarily right-lateralized frontoparietal visuospatial network (Rafal and Posner, 1987; Corbetta et al., 1995; Constantinidis and Steinmetz, 1996, 2005; Corbetta, 1998; Thiebaut de Schotten et al., 2011). Several studies have targeted the PPC with tDCS, and assessed behavioral impacts on tasks demanding spatial attention. In one study, anodal tDCS administered to the right PPC improved change detection in individuals with lower than ceiling performance, presumably due to enhanced spatial attention toward relevant areas of the task (Tseng et al., 2012). Another study demonstrated that anodal tDCS administered to the right PPC can ameliorate some spatial attention deficits shown by patients with left visuospatial neglect (Sparing et al., 2009). Additional studies demonstrate impacts of tDCS over the PPC on spatial orienting (Bolognini et al., 2010), spatial reorienting (Roy et al., 2015), and tests of lateralized spatial attention bias (de Tommaso et al., 2014). In one tACS study, gamma frequency stimulation over V1 was not shown to modulate spatial attention (though it did alter contrast perception; Laczó et al., 2012). In another, anti-phase gamma frequency stimulation over the left temporal and parietal cortex enhanced visual working memory, suggesting an impact on spatial attention (Tseng et al., 2016). Additional regions implicated in spatial attention, including the superior temporal sulcus, frontal eye fields, anterior cingulate and thalamic nuclei, have not been directly targeted by tDCS or tACS, or have been targeted but not in a manner related to spatial attention.

### Spatial Perception

Spatial perception involves perceiving and comprehending spatial information, particularly with regard to the body’s orientation (Loomis and Philbeck, 2008). This includes perceiving spatial relationships among objects, and your position relative to those relationships. Several studies have demonstrated that spatial perception engages areas of the PPC, most notably the right inferior parietal lobule, the middle and inferior frontal gyri, and the superior temporal gyrus (Andersen et al., 1985; Andersen, 1987; Woldorff et al., 1999; Ellison et al., 2004; Straube and Chatterjee, 2010). Very few studies have examined tES influences on spatial perception. In one, tDCS of the PPC altered the perception of object location, with mislocalization biased in the direction contralateral to stimulated hemisphere (Wright and Krekelberg, 2014). Stimulating the right PPC also induces polarity-specific modulation of spatial information reliance during causality inferencing (Straube et al., 2011). Research using tDCS or tACS to target the middle and inferior frontal gyri, and superior temporal gyrus, has not examined influences on spatial perception tasks.

### Mental Rotation

Mental rotation involves mental spatial transformations of objects around one or more axes of rotation. The seminal mental rotation task involves comparing two three-dimensional objects, mentally rotating one object to match or mismatch a reference object (Shepard and Metzler, 1971). An abundance of lesioning and functional neuroimaging studies has demonstrated the importance of the parietal cortex in mental rotation, with some studies suggesting a relatively right-lateralized mechanism (Ratcliff, 1979; Deutsch et al., 1988; Cohen et al., 1996; Tagaris et al., 1996, 1997; Richter et al., 1997; Gauthier et al., 2002; Jordan et al., 2002). Given the more general engagement of the prefrontal cortex in working memory and executive control tasks (D’Esposito et al., 1998), perhaps not surprisingly this region has been implicated in maintaining goals and monitoring and updating spatial relations during mental rotation (Cohen et al., 1996). Indeed targeting the dorsolateral prefrontal cortex with tDCS modulates performance on spatial working memory tasks in general (Alencastro et al., 2017), though it does not appear to specifically influence mental rotation performance (Oldrati et al., 2018). To date, no studies have specifically examined tDCS or tACS targeting the parietal cortex and measuring behavioral outcomes on a mental rotation task, however, three related studies are worth mentioning. One study leveraged an implanted array of electrodes over the parietal cortex, demonstrating that high intensity (7–12 mA) superior parietal stimulation dramatically and selectively impaired mental rotation ability (Zacks et al., 2003). Second, online and offline transcranial alternating current stimulation (tACS) was recently found to benefit mental rotation performance, though the electrode montage used did not specifically target parietal areas (Kasten and Herrmann, 2017).

### Spatial Visualization

Spatial visualization involves complex, sequential manipulations of spatial information (Linn and Petersen, 1985; Kozhevnikov et al., 2007). It involves spatial attention and perception, and sometimes mental rotation, but extends these processes to multi-step spatial procedures (e.g., Rubik’s cube, paper folding) with multiple analytic strategies that can be adopted to develop a solution. A broad network of functionally connected brain regions is implicated in spatial visualization, particularly executive control and working memory regions (e.g., dlPFC, anterior cingulate), and regions implicated in spatial perception, attention, and mental rotation (e.g., posterior and superior parietal cortices; Sack et al., 2007; Watson and Chatterjee, 2012). Very few studies have examined tDCS or tACS influences on spatial visualization. In one study, tDCS centered over the dlPFC enhanced training of a mental paper folding task; however, this pattern only emerged when tDCS was administered online (rather than offline, before the task; Oldrati et al., 2018).

## tES and Navigation

Wayfinding involves deliberate navigation between waypoints in large-scale environments, and is one of the most complex and frequent tasks undertaken by humans. It is often distinguished from the motoric sequences underlying the navigation of well-learned routes, for instance from home to a workplace, in that it also involves developing and using spatial representations to support movement (Hartley et al., 2003). Successful wayfinding generally involves recognizing places, learning sequences, identifying decision points and making decisions and behavioral responses, developing associations among environmental features, transforming perspectives, and constantly relating the directly perceived environment with environmental knowledge and goal representations (Allen, 1999; Klippel, 2003; Montello, 2005; Wiener et al., 2009; Dudchenko, 2010).

Elements of spatial attention, perception, mental rotation, visualization and working memory are critical for supporting wayfinding, and people differ dramatically in their ability to find their way through complex environments (Hegarty and Waller, 2005). The diverse engagement of cognitive processes in wayfinding is reflected in the diversity of brain regions implicated in supporting these processes (Maguire et al., 1999; Burgess et al., 2002; Shelton and Gabrieli, 2002; Schinazi and Epstein, 2010), and the diversity of taxonomies devoted specifically to understanding the processes engaged during wayfinding (Siegel and White, 1975; Wiener et al., 2009; Chrastil, 2013). Only one study has examined tES influences on wayfinding (Brunyé et al., 2014). In that study, the authors targeted the right medial temporal lobe with a multielectrode tDCS montage and demonstrated no main effect of tDCS on virtual wayfinding performance. Targeting deep brain structures in medial temporal areas may not be feasible with tES; instead, researchers may find value in indirectly targeting these areas by stimulating nodes in functional neural networks.

## Functional Networks in Navigation

In the above typology of spatial processes, we focused primarily on focal brain regions underlying spatial performance, though stable functional networks have also been identified supporting several aspects of spatial processing. Byrne et al. (2007) described a dynamic neural model to characterize interactions among brain regions implicated in human navigation. The model distinguishes between an egocentric system (posterior parietal), allocentric system (medial temporal), and transformational (retrosplenial) system. Functional connectivity analyses have identified at least three functional pathways involved in communicating among these systems (Kravitz et al., 2011):

1. The *parieto-prefrontal pathway* connects lateral and ventral intraparietal, and middle and medial superior temporal lobe areas, to prefrontal regions, and is involved in spatial attention including the initiation and control of eye movements, and top-down executive control of visuospatial working memory processing (Xu and Chun, 2006).
2. The *parieto-premotor pathway* consists of two parallel projections, one connecting the ventral intraparietal and dorsal premotor cortices, and the other connecting the medial intraparietal and ventral premotor cortices, both engaged in initiating and controlling several visually-directed actions (e.g., reaching for and grasping objects) using parietal object representations (Blangero et al., 2009; Reichenbach et al., 2014).
3. Finally, a *parieto-medial temporal pathway* connects the caudal inferior parietal lobe (cIPL) and a range of areas including retrosplenial cortex (RSC) and PCC, and secondarily to the hippocampus and parahippocampus (Rushworth et al., 2006). Inferior parietal areas have been implicated in a range of navigation-relevant functions, including representing distant space in world- and object-centered frame of reference, egocentric heading direction, and egocentric distance (Brotchie et al., 1995; Crowe et al., 2005; Chafee et al., 2007; Harvey et al., 2012), suggesting importance for real-world navigation. Concordant with intermediary roles between posterior parietal and medial temporal regions, the PCC and RSC have been implicated in translating between egocentric (parietal) and allocentric (medial temporal) representations of space, and in relating optic flow to heading direction and movement toward goals (Vogt et al., 1992; Burgess, 2008; Epstein, 2008; Sherrill et al., 2015; Boccia et al., 2016; Wiener et al., 2016).

Research continues to better define the anatomical and functional links between brain regions implicated in spatial processing and navigation. One outcome of this research is affording better understandings of how tES may prove tractable for modulating brain circuits engaged in spatial processing.

## Leveraging Functional Connectivity with tES

Few studies have examined the influence of modulating superficial tES targets on more distributed neural networks. In two such studies, tDCS of the left dlPFC altered resting-state connectivity in several functional networks, including the default mode network, frontal-parietal network, and self-referential network (Keeser et al., 2011; Peña-Gómez et al., 2012). In the spatial processing domain, two related studies administered tDCS over the parietal cortex and found altered functional connectivity between this region and the prefrontal cortices and several subcortical regions, both during a virtual navigation task (Hampstead et al., 2014) and during resting state (Krishnamurthy et al., 2015). No reliable impacts on behavior were found, however, in this research the stimulated electrode site (Pz) sits over bilateral superior parietal regions rather than a lateral inferior parietal lobule.

**Figure 1:**
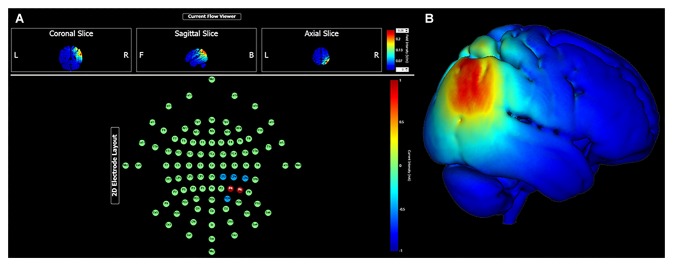
Transcranial direct current stimulation (tDCS) targeting the right caudal inferior parietal lobule (angular gyrus) with 2.0 mA current intensity. Panel **(A)** shows electrode montage and current flow in coronal, sagittal and axial views. Panel **(B)** shows electrical field intensity overlaid onto a standard MNI head model (MNI 152).

Continuing research may find value in specifically targeting the parieto-medial temporal and parieto-prefrontal pathways. High fidelity head models that predict current propagation can be used to maximize current density at relatively superficial cortical targets, such as the right inferior parietal lobule. For instance, using the HD Targets finite element model developed by Soterix Medical Incorporation (New York, NY, USA), Figure 1 demonstrates predicted current density with a multielectrode array targeting the cIPL (i.e., angular gyrus). This montage uses two anodes at locations P6 (1.0 mA) and P4 (1.0 mA), and three cathodes at locations CP2, CP4, CP6, and PO4 (0.5 mA each). With 2.0 mA total current, maximum electrical field intensity at target is approximately 0.22 V/m. Given functional connectivity between the cIPL and the RSC, PCC, hippocampus and parahippocampus, targeted neuromodulation of the relatively lateral cIPL region might be expected to modulate several aspects of spatial processing with implications for large-scale navigation performance, for instance egocentric and allocentric perspective switching and integration, and maintaining orientation relative to goal locations.

Behavioral outcomes related to parieto-medial temporal pathway might be dissociated with outcomes of targeting the parieto-prefrontal pathway. Specifically, targeting lateral and ventral intraparietal areas might be expected to impact visuospatial spatial working memory performance, whereas targeting the cIPL may not. These types of dissociations between stimulation locations, stimulation conditions (e.g., tDCS, tACS), and behavioral outcomes can help elucidate behavioral influences of each pathway, and reveal methods for altering spatial performance. Furthermore, as research reveals the oscillatory dynamics of the parieto-medial temporal pathway, frequency-specific tACS might also prove valuable for modulating network resonance and behavioral outcomes (Ali et al., 2013; Marshall and Binder, 2013).

## Conclusion

People differ dramatically in spatial abilities (Hegarty and Waller, 2005), and identifying reliable methods for enhancing or accelerating spatial skills education and training may prove valuable in both healthy and clinical populations. For instance, body- and world-centered spatial visualization skills are fundamental to many work-related domains, especially science, technology, engineering and mathematics (STEM) disciplines (Sorby, 1999; Titus and Horsman, 2009; Taylor and Hutton, 2013; Uttal et al., 2013; Burte et al., 2017). Future research might find value in using tES to selectively upregulate networks engaged in successful spatial thinking. This research will be enabled by at least three specific research areas. First, better defining functional connectivity between cortical and subcortical brain regions during spatial processing and navigation, and how these networks might vary in structure and function across individuals. Second, identifying how tES modulates cortical and network activity, and how these dynamics might vary over time and across individuals (Krause et al., 2013). Third, advances in finite element modeling that include customized (i.e., individualized) cortical targets will afford specificity and reliability of stimulation protocols (Radman et al., 2009), and possibly enhance real-world behavioral outcomes.

## Author Contributions

TTB conceived the review and prepared the manuscript.

## Conflict of Interest Statement

The author declares that the research was conducted in the absence of any commercial or financial relationships that could be construed as a potential conflict of interest.
